# Chemical Polysialylation and In Vivo Tetramerization Improve Pharmacokinetic Characteristics of Recombinant Human Butyrylcholinesterase-Based Bioscavengers

**Published:** 2015

**Authors:** S. S. Terekhov, I. V. Smirnov, O. G. Shamborant, T. V. Bobik, D. G. Ilyushin, A. N. Murashev, I. A. Dyachenko, V. A. Palikov, V. D. Knorre, A. A. Belogurov, N. A. Ponomarenko, E. S. Kuzina, D. D. Genkin, P. Masson, A. G. Gabibov

**Affiliations:** Shemyakin-Ovchinnikov Institute of Bioorganic Chemistry of the Russian Academy of Sciences, Miklukho-Maklaya Str., 16/10, Moscow, 117997, Russia; Branch of the Shemyakin-Ovchinnikov Institute of Bioorganic Chemistry of the Russian Academy of Sciences, Prospekt Nauki, 6, Pushchino, Moscow Region, 142290, Russia; Kazan’ Federal University, Kremlevskaya Str., 18, Kazan’, Republic of Tatarstan, 420000, Russia; Institute of Gene Biology of the Russian Academy of Sciences, Vavilova Str., 34/5, Moscow, 119334 , Russia; Pharmsynthez, Krasnogo Kursanta Str., 25zh, Saint Petersburg, 197110, Russia

**Keywords:** bioscavenger, biopharmaceutical, biodistribution, butyrylcholinesterase, polysialylation, pharmacokinetics, in vivo tetramerization

## Abstract

Organophosphate toxins (OPs) are the most toxic low-molecular compounds. The extremely potent toxicity of OPs is determined by their specificity toward the nerve system. Human butyrylcholinesterase (hBChE) is a natural bioscavenger against a broad spectrum of OPs, which makes it a promising candidate for the development of DNA-encoded bioscavengers. The high values of the protective index observed for recombinant hBChE (rhBChE) make it appropriate for therapy against OP poisoning, especially in the case of highly toxic warfare nerve agents. Nevertheless, large-scale application of biopharmaceuticals based on hBChE is restricted due to its high cost and extremely rapid elimination from the bloodstream. In the present study, we examine two approaches for long-acting rhBChE production: I) chemical polysialylation and II) *in-vivo *tetramerization. We demonstrate that both approaches significantly improve the pharmacokinetic characteristics of rhBChE (more than 5 and 10 times, respectively), which makes it possible to use rhBChE conjugated with polysialic acids (rhBChE-CAO) and tetrameric rhBChE (4rhBChE) in the treatment of OP poisonings.

## INTRODUCTION

Organophosphate toxins (OPs), despite their more than 150-year history, remain some of the most topical objects in modern toxicology. OP toxins represent several classes of organophosphorus compounds that irreversibly inhibit human acetylcholinesterase (hAChE). The inhibition of acetylcholinesterase, in turn, leads to the development of the salivation, lacrimation, urination, diaphoresis, gastrointestinal upset, and emesis (miosis) syndrome (SLUDGE(M)). Acute poisoning leads to convulsions, permanent brain damage, respiratory arrest, and death. Currently, OP victims (about 260,000 per year) are mainly suicides. This is especially true for the Western Pacific Region, which accounts for approximately 50% of the total number of suicide attempts [1]. Also, poisonings by organophosphate pesticides often occur among farmers. In addition, there is a potential threat in military use of neuroparalytic warfare poisonous agents or their use in terrorist attacks. The convenient management of OP poisoning [2] includes combined therapy with muscarinic receptor antagonists (usually atropine) and acetylcholinesterase reactivators (pralidoxime or obidoxime). Unfortunately, this therapy is not a panacea; it does not increase survival in poisoning by organophosphorus pesticides [3] and also does not prevent permanent brain damage.

An alternative approach for the treatment of OP poisonings is the application of bioscavengers – biomolecules binding and inactivating OPs [4-7]. Human butyrylcholinesterase is a natural bioscavenger (suicide inactivator) in OP poisoning [8]. hBChE that has a large active site cavity and a unique similarity to hAChE inactivates a wide range of OPs, often more effectively than hAChE [9]. The use of hBChE in the therapy of OP poisonings not only improves the survival rate, but also obviates the side effects of long-term OP poisoning, including permanent brain damage [10]. Despite the obvious advantages, the application of hBChE in the treatment of OP poisonings is very limited by the high cost of hBChE-based drugs and the extremely rapid elimination (τ_1/2_ ≈ 2 min) of monomeric and dimeric recombinant hBChE (rhBChE) forms from the circulation [11]. Thus, the main efforts in the development of an effective therapeutic drug have focused on enhancing rhBChE production [12] and improving the pharmacokinetics of rhBChE-based drugs through chemical conjugation with polyethylene glycol [13-16] and polysialic acids (CAO) [17], or fusing rhBChE with human serum albumin [18]. Recently, we showed [19] that a high production level and, at the same time, a significant improvement in the pharmacokinetic characteristics of rhBChE can be achieved by *in vivo *rhBChE tetramerization. We demonstrated that simulation of natural rhBChE tetramerization [20] in a conventional expression system of the CHO cell line provides effective biotechnological production of a tetrameric rhBChE-based (4rhBChE) biopharmaceutical. 4rhBChE produced by* in vivo *tetramerization had pharmacokinetic characteristics (τ_1/2_ 32 ± 1.2 h, MRT 43 ± 2 h) similar to those of a tetrameric hBChE drug obtained from human blood plasma [21].

The purpose of this study was to investigate the possibility of further enhancement in the pharmacokinetic characteristics of the 4rhBChE drug by means of chemical polysialylation and to determine the effect of polysialylation on the biodistribution profile of rh- BChE-based biopharmaceuticals in mouse models.

## MATERIALS AND METHODS

**4rhBChE-based biopharmaceuticals used in the study**

4rhBChE was produced in CHO-K1 cells transfected with the construct pFUSE PRAD-F2A-BChE. The cells simultaneously expressed genes of the tetramerization peptide (PRAD-peptide) and human butyrylcholinesterase [19]. rhBChE was obtained as a mixture of oligomers [17] with a predominant content of the dimeric form. BChE was successively purified by affinity chromatography on a XK10/50 column (GE Healthcare, USA) packed with a procainamide-Sepharose sorbent and ion exchange chromatography on a MonoQ 5/50 column (GE Healthcare, USA). According to polyacrylamide gel electrophoresis with Coomassie staining and staining for the specific butyrylcholinesterase activity by the Karnovsky and Roots method [22], the protein purity was greater than 95%.

**Chemical polysialylation of rhBChE biopharmaceuticals**

rhBChE samples were chemically conjugated to oxidized polysialic acids with a mean molecular weight of 24 kDa (Xenetic Biosciences) by reductive amination according to [17, 23]. The conjugation was performed in 0.1 M potassium phosphate buffer, pH 6.9, with the molar rhBChE : CAO ratio being 1 : 50 per the rhBChE monomer. The final NaBH3CN concentration was 3 mg/mL. The reaction was conducted at 25 °C for 48 h. The resulting rhBChE-CAO conjugate was purified from reaction by-products by repeated dialysis using Amicon Ultra-15 30K concentrators (Millipore, USA). The efficiency of modification was determined by electrophoresis in 8% polyacrylamide gel (with SDS, but without β-mercaptoethanol). The concentration of active rhBChE was determined by the Ellman’s method [23] using 1 mM butyrylthiocholine iodide (Sigma) and 0.5 mM 5,5-dithiobis(2-nitrobenzoic acid) (Sigma) in 0.1 M potassium phosphate buffer, pH 7.0, at 25 °C. The formation of a reaction product, 5-thio-2-nitrobenzoic acid, was detected spectrophotometrically at a wavelength of 412 nm using a product molar absorption coefficient of 13,600 M^–1^ cm^–1^. The BChE concentration was evaluated based on the specific activity of 720 units of activity per 1 mg of pure BChE.

**Determination of the pharmacokinetic parameters of rhBChE biopharmaceuticals and rhBChE-CAO conjugates**

The concentration of rhBChE, rhBChE-CAO, 4rh- BChE, and 4rhBChE-CAO in blood plasma was determined using four groups of BALB/c mice, 18 animals each. Each group consisted of three subgroups, six animals each, for time intervals of 2 min–3 h (subgroup I), 1 h–3 days (subgroup II), and 1–8 days (subgroup III). BChE biopharmaceuticals were administered intravenously at a dose of 200 μg/mouse (subgroups I and II) and 500 μg/mouse (subgroup III). Blood samples were collected from the orbital sinus after 2, 5, 10, 15, and 30 min, 1, 2, 3, 6, 9, and 24 h, and 2, 3, 4, 5, 6, and 7 days after administration. The BChE concentration in mouse blood serum was determined based on the BChE activity according to the Ellman’s method [24]. The pharmacokinetic characteristics of the samples were obtained based on fitting the BChE elimination curve using the two-compartment model [17] with the SigmaPlot 12.5 software (Systat software).

**Profiling of the rhBChE and rhBChECAO conjugate biodistribution**

rhBChE and rhBChE-CAO samples were radiolabeled with 125I using chloramine-T at a dose of 106 cpm/mg. Labeled rhBChE and rhBChE-CAO samples were intravenously administered to BALB/c line mice (three groups of six animals for each drug) at a dose of 105 cpm/mouse. Mice were sacrificed after 0.5, 3, and 48 h, and samples of their blood and tissues were collected and weighed. Collected samples were measured using a WIZARD automated gamma counter (PerkinElmer). Accumulation in tissue was defined as a ratio of the organ specific radioactivity (cpm/g) to the blood specific radioactivity (cpm/mL) at a given time.

## RESULTS AND DISCUSSION

Major advances associated with the therapeutic application of rhBChE biopharmaceuticals were achieved for rhBChE chemically modified with polyethylene glycol. Previously, we demonstrated [17] that chemical polysialylation can be used as an alternative modification that repeatedly improves the pharmacokinetic characteristics of rhBChE. The pharmacokinetic characteristics of rhBChE-polysialic acid (rhBChE-CAO) conjugates are inferior to those of rhBChE-polyethylene glycol conjugates [12, 13, 15], but the former have a significant advantage in biodegradability over the latter. In this study, we compared the pharmacokinetic characteristics of rhBChE-CAO and 4rhBChE biopharmaceuticals without chemical modification [19], as well as evaluated the effect of chemical polysialylation on the 4rhBChE-CAO conjugate pharmacokinetics.

**Fig. 1:**
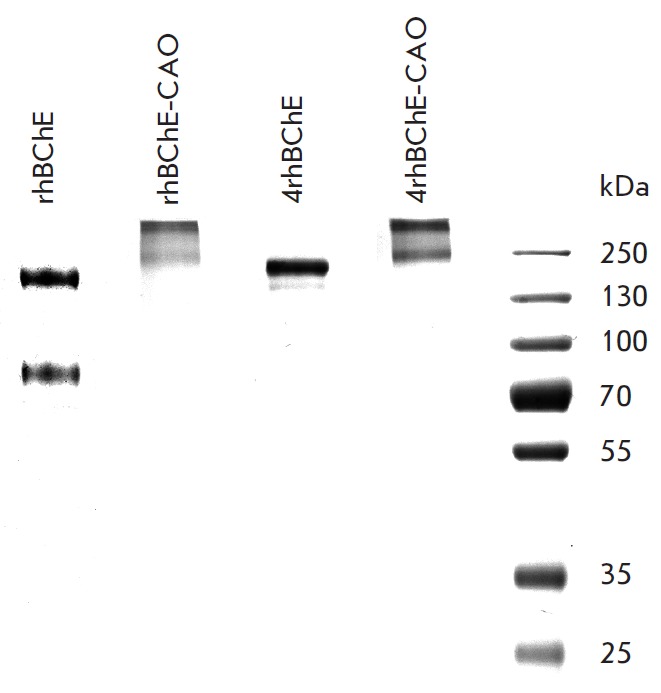
Electrophoretic analysis of the studied BChE-based biopharmaceuticals and their conjugates with polysialic acids (CAO). Separation was carried out in 8% PAGE under non-reducing conditions, followed by Coomassie R-250 staining. An rhBChE sample is present as a mixture of monomeric, dimeric, and tetrameric forms; a 4rhBChE sample is present exclusively in the tetrameric form. Polysialylated BChE samples obviously have higher molecular weights, but broad bands; this effect was described for chemically polysialylated proteins [17]

Polysialylation of rhBChE and 4rhBChE proceeds with efficiency of over 95% and leads to the formation of high-molecular-weight products with a modification degree of about six CAO molecules per BChE monomer (*Fig. 1*). The resulting rhBChE-CAO and 4rhBChE-CAO conjugates had low toxicity and did not cause the death of the experimental animals after an intravenous injection at a dose of up to 1,500 mg/kg, which, in turn, may indicate a potential for increasing the protective index by more than an order of magnitude relative to the data obtained previously for the warfare agent VR [17].

**Table:** Pharmacokinetic characteristics of BChE biopharmaceuticals

Biopharmaceutical	Pharmacokinetic parameters		
	τ_1/2distr._, h	τ_1/2el._, h	MRT, h
rhBChE	0.2±0.1	3±1	3±1.6
rhBChE-CAO	0.3±0.1	14±2	19±3
4rhBChE	2.4±0.3	33±2	43±4
4rhBChE-CAO	0.8±0.2	19±2	27±3

**Fig. 2:**
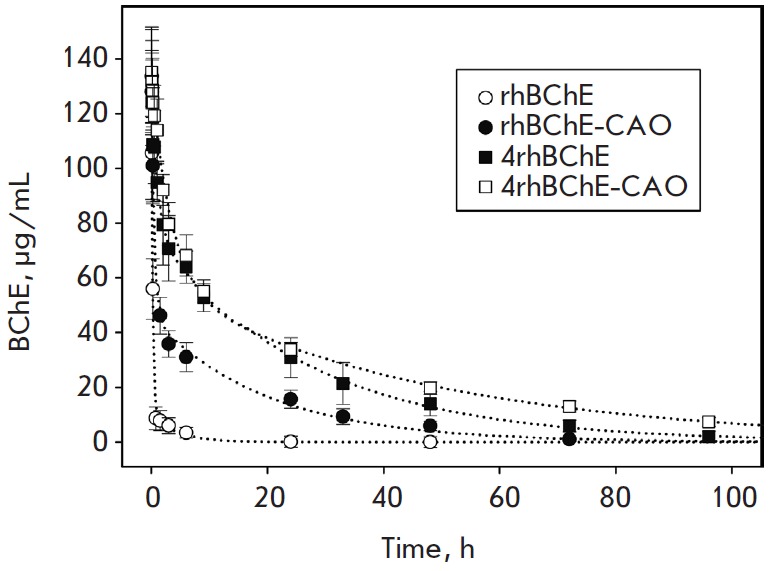
Pharmacokinetics of the elimination of BChE-based biopharmaceuticals from the circulation after an intravenous injection. To evaluate BChE biopharmaceutical concentrations in blood plasma, 4 groups of BALB/c mice, 18 animals each, were used. The animals were intravenously administered with rhBChE, rhBChE-CAO, 4rhBChE, and 4rhBChE-CAO at doses of 200 and 500 μg/mouse. The BChE concentration in mouse blood serum was determined by the Ellman’s method. The pharmacokinetic characteristics of the biopharmaceuticals were obtained by fitting the drug elimination curve to a two-compartment model

To evaluate the pharmacokinetic characteristics of the produced BChE drugs, a mouse model of intravenous drug administration and determination of the residual butyrylcholinesterase activity in blood serum were used. The endogenous BChE activity level in mouse blood serum was 2.0 ± 0.5 μg/mL, which enabled a highly accurate estimation of the administered drug’s concentration. *Fig. 2* shows the elimination curves for the studied biopharmaceuticals. It is obvious that practical application of rhBChE without modification is largely complicated by an extremely rapid elimination of rhBChE from the circulation. The modification with polysialic acids provides a more than 5-fold enhancement of the rhBChE pharmacokinetic characteristics (*Table*), which significantly extends the range of its therapeutic applications and enables its use for the prevention of OP poisoning. At the same time, 4rh- BChE has characteristics that are more than twice better than those of the rhBChE-CAO conjugate; thereby, 4rhBChE has the longest clearance time from circulation among the studied drugs. The biotechnological production of 4rhBChE is similar to that of rhBChE and is much more economically feasible than the production of the rhBChE-CAO conjugate due to the absence of modification (which uses a 50-fold excess of CAO) and purification stages. At the same time, one could expect that polysialylation of 4rhBChE would lead to a further enhancement of the pharmacokinetic characteristics of 4rhBChE, but this does not occur. The elimination pharmacokinetics of 4rhBChE-CAO and 4rhBChE is almost identical on the first day; at lengthier times, 4rhBChE-CAO is eliminated faster than unmodified 4rhBChE. Therefore, chemical polysialylation repeatedly enhances the pharmacokinetic characteristics of monomeric and dimeric rhBChE forms but does not improve the 4rhBChE pharmacokinetics. Since hBChE is present in human blood plasma only in the tetrameric form, which ensures its long-term circulation, and chemical polysialylation of 4rhBChE does not lead to an improvement in the pharmacokinetic characteristics of 4rhBChE-CAO compared to those of 4rhBChE, we may assume that the longer circulation of 4rhBChE is primarily associated with no increase in the hydrodynamic radius of 4rhBChE. Apparently, 4rhBChE complex formation leads to masking protein domains responsible for the rapid elimination of rhBChE.

**Fig. 3:**
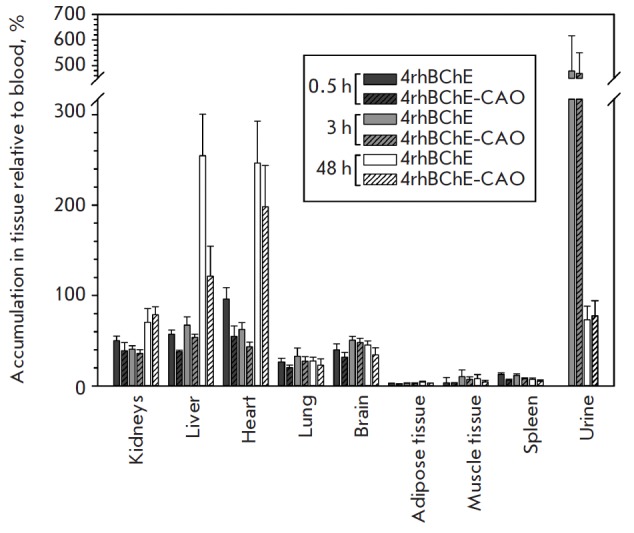
Biodistribution profiles for ^125^I-radiolabeled 4rhBChE and 4rhBChECAO 0.5, 3, and 48 h after their intravenous administration. 125I-labeled samples were obtained according to the standard protocol using chloramine T and purified by gel filtration. BALB/c mice (3 groups of 6 animals each per protein) were used in the experiments. Samples were injected into the tail vein at a dose of 105 cpm/mouse. The animals were euthanized after 0.5, 3, or 48 h. The appropriate organs were isolated, weighed, and used for the radiological analysis on a WIZARD automatic gamma counter (PerkinElmer). Accumulation in tissue was defined as a ratio of the organ specific radioactivity (cpm/g) to the blood specific radioactivity (cpm/mL) at a given time

To study the impact of chemical polysialylation on the profile of biodistribution and accumulation of 4rh- BChE drugs, experiments with the drugs labeled with the ^125^I radioisotope were conducted. 4rhBChE and 4rhBChE-CAO were administered intravenously, and their accumulation in different compartments was analyzed after 0.5, 3, and 48 h relative to the appropriate radioactivity of the blood samples (*Fig. 3*). No specific accumulation of 4rhBChE and 4rhBChE-CAO in organs occurs within the first 3 h, but pronounced urinary excretion is observed, which is apparently associated with the biodegradation products. Accumulation of the drugs in the kidneys and liver occurs 48 h after and is significantly more pronounced for 4rhBChE. As mentioned earlier, the elimination pharmacokinetics of 4rhBChE and 4rhBChE-CAO are very similar within the first 24 h, which also manifests itself in the similarity of biodistribution profiles. At the same time, after 48 h, the pharmacokinetic properties of 4rhBChE are better than those of 4rhBChE-CAO. Apparently, this is related to the more pronounced 4rhBChE accumulation in the kidneys, which leads to a reduction in its excretion rate. Along with this, an extremely low rhBChE level in the brain, as well as adipose and muscle tissue, should be noted. Residual radioactivity in these compartments is apparently associated with vascularization, which indicates a limited penetration capability typical of rhBChE.

## CONCLUSIONS

The purpose of this work was to study the impact of alternative approaches for increasing the duration of rhBChE circulation on rhBChE-based drugs pharmacokinetics. The improvement in the pharmacokinetic characteristics of rhBChE-CAO compared to those of rhBChE is apparently associated with an increase in the hydrodynamic radius of rhBChE-CAO and masking of the rhBChE domains (in particular, the C-terminal domain of hBChE) responsible for tetramerization by CAO molecules. A similar effect can be achieved through the production of 4rhBChE. The use of 4rh- BChE is an economically attractive alternative to biopharmaceuticals on the basis of modified rhBChE, because 4rhBChE allows one to achieve better pharmacokinetic parameters compared to those of rhBChE and rhBChE-CAO. Chemical modification of 4rhBChE by polysialic acids, in turn, does not lead to further improvement in the 4rhBChE-CAO pharmacokinetics, which may indicate the existence of additional natural mechanisms of 4rhBChE stabilization. At the same time, it should be recognized that further optimization of the polysialylation reaction, complete standardization of the chemical modification process, and use of recently proposed genetic expression constructs [19] may again bring rhBChE-CAO to the fore among potential OP bioscavengers.

The low toxicity of 4rhBChE-based biopharmaceuticals extends the opportunity for bioscavenger application in the treatment of OP poisonings. At the same time, it should be noted that application of 4rhBChE biopharmaceuticals is limited by the need to administer stoichiometric amounts of the enzyme with respect to OP. This fact, in turn, leads to the possibility of the protective index of 4rhBChE therapy (ratio of LD_50_ for animals after treatment to LD_50_ for animals without treatment) being high only in the case of warfare agents (i.e., highly toxic agents with low LD_50_). Further improvement of 4rhBChE-based biopharmaceuticals should be obviously associated with the creation of catalytic bioscavengers – enzymes that catalytically inactivate OPs. This will repeatedly reduce the therapeutic dose and extend the capabilities of this therapy for pesticide poisoning, because the high LD_50_ value in this case leads to the necessity of introducing an excessively high amount of the 4rhBChE drug. At the same time, the transition to catalytic bioscavengers should be associated with rapid and effective (k_2_/K_M_ ≈ 10^7^ M^–1^ min^–1^) OP elimination [13], which is particularly important in the treatment of OP poisonings [25, 26]. Of particular importance for developing a catalytic bioscavenger will be the issue of standardizing a highly productive clone which is economically viable for launching production, as well as its possible certification to FDA requirements. In the case of the stoichiometric rhBChE-based bioscavengers discussed in this article, the latter condition is absolutely feasible.

## Abbreviations

OPs: organophosphates
hAChE: human acetylcholinesterase
4rhBChE: tetrameric recombinant human butyrylcholinesterase
4rhBChE-CAO: chemically polysialylated 4rhBChE
MRT: mean residence time
PRAD: proline-rich attachment domain
CAO: Colominic (polysialic) Acid Oxidized
